# Evaluating the effectiveness of an educational curriculum on physician comfort in screening and managing eating disorders in the outpatient pediatric setting

**DOI:** 10.3389/fped.2026.1897533

**Published:** 2026-08-27

**Authors:** Xhesika Begaj, Elena Lorenzana, Stephanie Marcy, Colleen Kraft

**Affiliations:** 1 Children’s Hospital of Los Angeles, Los Angeles, CA, United States; 2 University of Southern California, Los Angeles, CA, United States

**Keywords:** adolescent medicine, eating disorder (ED), educational curriculum, outpatient care, physician comfort, screening tool development

## Abstract

**Background:**

Eating disorders are among the most prevalent chronic conditions affecting adolescents, yet remain underdiagnosed in outpatient pediatric settings. Physician discomfort with screening and management contributes to this gap, particularly in underserved communities. This study evaluated whether a structured educational curriculum paired with an EMR-integrated clinical decision support tool improves physician comfort with eating disorder screening and management, and increases screening behavior, in an outpatient pediatric clinic.

**Methods:**

This pre/post study (IRB CHLA-24-00118) was conducted at a CHLA AltaMed outpatient pediatric clinic. Physicians completed surveys at baseline, three months, and six months following implementation of the curriculum and screening tool. Comfort was self-rated on a 1–10 scale across three domains: screening for eating disorders, managing anorexia nervosa/restrictive eating disorders, and managing binge eating disorders. Of 35 enrolled physicians, 11 matched pairs completed both the baseline and six-month surveys and were included in the primary analysis. Wilcoxon signed-rank tests were used to assess change, with Hodges–Lehmann median estimates and bootstrap 95% confidence intervals.

**Results:**

Physician comfort improved significantly across all three domains (all *p* < 0.05), with a median change of +3.0 points each: screening comfort rose from 5.0 to 8.0 (*p* = 0.0010), anorexia/restrictive eating disorder management comfort rose from 3.0 to 6.0 (*p* = 0.0164), and binge eating management comfort rose from 2.0 to 7.0 (*p* = 0.0090). Self-reported screening rates increased from a median of 5% to 45% of patients (+40 percentage points; 95% CI 10–60; *p* = 0.0039). The percentage of patients screening positive did not change significantly (*p* = 0.0625), likely reflecting a floor effect. At six months, 65% of physicians had used the EMR-integrated screening tool and 87% rated the curriculum as beneficial.

**Conclusion:**

A structured educational curriculum combined with an EMR-integrated screening tool was associated with significant improvements in physician comfort and self-reported screening behavior for eating disorders in an outpatient pediatric setting. These findings support the feasibility of low-resource, locally developed interventions to close a persistent gap in physician training.

## Introduction

Eating disorders (EDs) are among the most prevalent and consequential chronic conditions affecting adolescents. They represent the third most common chronic illness in this age group, yet they continue to be underdiagnosed in the outpatient setting (1). This gap in detection carries significant clinical consequences: anorexia nervosa (AN) has the highest mortality rate of any psychiatric disorder, and even sub-threshold presentations are associated with serious medical morbidity (1).

Prior to the publication of the Diagnostic and Statistical Manual of Mental Disorders, Fifth Edition (DSM-5), a large proportion of patients presenting with disordered eating were classified under the catch-all category of “eating disorder not otherwise specified” (EDNOS). These patients often presented with some, but not all, features of AN or bulimia nervosa (BN), yet experienced medical complications at rates comparable to those with full-threshold diagnoses (2). The expanded and revised diagnostic criteria introduced in DSM-5 were intended to address this diagnostic gap and improve recognition of the full spectrum of disordered eating.

Despite improved diagnostic frameworks, eating disorders continue to evade detection in many outpatient pediatric settings. Earlier identification and intervention are associated with better recovery outcomes, fewer medical complications, and an improved ability to address the underlying factors contributing to disordered eating (2). Pediatric outpatient visits, particularly well-child checks, represent a critical opportunity for routine screening, yet standardized screening practices remain inconsistent across providers.

Disparities in access to eating disorder care compound this problem. Patients with public insurance are approximately one-third as likely to receive treatment compared to those with private insurance, and Latinx and Asian patients are approximately half as likely to access care (3). Children's Hospital Los Angeles (CHLA) serves a predominantly publicly-insured, underserved population through its AltaMed clinics, making improvements in early detection especially important for addressing health equity in this community.

Physician comfort and confidence also play an important role in whether screening occurs. Educational interventions targeting provider knowledge and self-efficacy have shown promise in improving clinical behaviors in other areas of pediatric practice; however, structured curricula focused specifically on eating disorder screening and management for outpatient pediatric providers remain limited. This study evaluates the effectiveness of an independent educational curriculum, paired with a standardized screening tool, in improving physician self-reported comfort with eating disorder screening and management, and examines its impact on screening rates over time. We hypothesized that (1) the educational intervention would significantly increase physician self-reported comfort with screening for and managing patients with eating disorders, and (2) the introduction of a standardized screening tool would increase the number of patients screened for eating disorders in the outpatient setting.

## Materials and methods

### Study design

This was a single-center, longitudinal cohort study using a pre-test/post-test design. The study was conducted at CHLA AltaMed, a general pediatrics outpatient clinic affiliated with Children's Hospital Los Angeles. The study was approved by the CHLA Institutional Review Board (IRB #CHLA-24-00118).

### Participants

Eligible participants included pediatric residents, general pediatric medicine fellows, and attending physicians based at CHLA AltaMed for their general pediatrics clinic. Physicians based at other AltaMed locations were excluded, as were those who did not engage with any component of the educational curriculum (i.e., did not watch any lectures, use the available clinical handbooks or use the eating disorder screening tool). Participation was entirely voluntary, and all physicians were able to engage with the educational curriculum regardless of whether they enrolled in the research study. Recruitment was conducted via email and in-person outreach at the clinic site by members of the research team.

### Educational intervention

An independent eating disorder educational curriculum was developed and made available to all CHLA AltaMed physicians, irrespective of study enrollment. The curriculum was developed by the study's principal investigator (XB) with input from the study's clinical mentors, and lecture content was delivered in partnership with Equip Health, a national eating disorder treatment organization. Completion of the curriculum was not mandatory for study enrollment; attendance at individual lecture sessions and use of the clinical handbooks were not formally tracked, and engagement was assessed via participant self-report. Participants had the opportunity to provide feedback on the curriculum through free-response items on the six-month follow-up survey (Survey 3). The curriculum included five one-hour lectures delivered both in-person at CHLA and pre-recorded and shared virtually. Topics covered in the lectures included identification and conversation approach, red flag signs, atypical presentations of eating disorders and myth busters, how to develop a treatment plan and when to consider medication. Ten copies of The Treatment of Eating Disorders: A Clinical Handbook were also purchased and placed in the physician workrooms at CHLA AltaMed for easy reference.

A standardized eating disorder screening tool was developed using DSM-5 diagnostic criteria and clinical features outlined in the American Academy of Pediatrics (AAP) clinical report, *Identification and Management of Eating Disorders in Children and Adolescents* (4). The dot phrase was built and validated in the EMR test environment by the study team prior to launch. The tool was entered into the Epic electronic medical record (EMR) system as a dot phrase (.EDscreen), allowing physicians to populate screening questions directly into clinical notes. Participants and non-participants were encouraged to use the tool during all well-child visits for patients aged 12 years and older, as well as during sick or follow-up visits when clinical concern for disordered eating was present.

### Surveys and data collection

Data were collected via three online surveys administered through Google Forms at baseline (Survey 1), three months (Survey 2), and six months (Survey 3) after the start of the study. Each survey took approximately five minutes to complete. To link responses across time points, participants were assigned a unique numeric code prior to completing the baseline survey. Codes were distributed via email after participants completed an initial enrollment form. Participants entered their unique code on each subsequent survey, allowing tracking of responses without collection of personally identifying information. Codes were tracked using a private spreadsheet accessible only to the four members of the research team.

Survey 1 collected baseline information on physician self-reported comfort levels with screening for and managing patients with eating disorders, current screening methods, estimated number and percentage of patients screened for disordered eating, and estimated number and percentage of patients diagnosed with an eating disorder. Surveys 2 and 3 repeated these questions to enable within-subject comparisons across time points. Survey items were developed by the study team specifically for this project and were not derived from a previously validated instrument.

### Outcomes

The primary outcome was change in physician self-reported comfort levels in screening for and managing patients with eating disorders at three and six months compared to baseline. Secondary outcomes included the estimated number and percentage of patients screened for eating disorders per physician and the estimated number and percentage of patients who screened positive for disordered eating over the study period.

### Statistical analysis

Statistical analyses were conducted in consultation with CHLA biostatisticians. Physician self-reported comfort levels were measured on a 1–10 numeric scale across three domains: screening for eating disorders, managing anorexia nervosa and restrictive eating disorders, and managing binge eating disorders. To assess change in comfort from baseline to the six-month follow-up, Wilcoxon signed-rank tests were used for each domain, with each participant serving as their own control. This non-parametric approach was selected to enable consistent reporting of Hodges–Lehmann (HL) median estimates and rank-based effect sizes. One participant was excluded from all analyses after self-reporting non-attendance at the educational curriculum; analyses were therefore restricted to participants who completed both the baseline survey (Survey 1) and the six-month follow-up survey (Survey 3), could be matched via their unique study identifier, and attended the curriculum (*n* = 11). The three-month interim survey (Survey 2) was completed by only seven participants and was therefore underpowered for paired analysis; those responses were not included in the primary statistical analysis. Effect sizes are reported as r (rank-biserial correlation). Statistical significance was defined as *p* < 0.05. Ninety-five percent confidence intervals for median estimates are bootstrap-based (10,000 iterations). Missing data were handled via complete-case analysis: participants who did not complete both the baseline and six-month surveys were excluded from the paired analysis rather than imputed, given the small overall sample size. No correction for multiple comparisons was applied across the three comfort domains or two secondary outcomes, consistent with the exploratory, hypothesis-generating nature of this pilot study; results should be interpreted with this in mind.

## Results

### Participant characteristics

Of 35 physicians enrolled in the study, 25 (71.4%) completed the baseline survey (Survey 1), 7 (20.0%) completed the three-month survey (Survey 2), and 23 (65.7%) completed the six-month follow-up survey (Survey 3). After matching respondents across both surveys using unique study identifiers, 11 participants were included in the paired analysis for comfort outcomes. The remaining participants either completed only the baseline survey (*n* = 10) or only the follow-up survey (*n* = 8) and were therefore excluded from the primary paired analysis. Demographic characteristics such as training level (resident, fellow, or attending) and years in practice were not collected as part of the study surveys, precluding subgroup analysis by training status.

### Primary outcome: physician comfort levels

Table 1 shows the self-reported comfort in screening and managing eating disorders at baseline (Survey 1) and at six-month follow-up (Survey 3). At baseline, physicians reported only average comfort with screening and low comfort with management of eating disorders. On a 10-point Likert scale, median comfort scores were 5.0 (IQR 4–6) for screening, 3.0 (IQR 2–4) for managing anorexia nervosa/restrictive eating disorders, and 2.0 (IQR 2–4) for managing binge eating disorders. Following intervention, participants demonstrated statistically significant improvements (*p* < 0.05) in all comfort domains with an estimated median change of +3.0 points across all three domains with large effect sizes. The narrow post-intervention IQR for screening comfort (8–9) suggests convergence toward uniformly high comfort among participants.

**Table 1:** Physician self-reported comfort scores: pre- vs. post-Intervention (*n* = 11, Wilcoxon signed-rank).

Domain	Median (S1) [IQR]	Median (S3) [IQR]	Median Change (95% CI)	*p*-value
Screening for eating disorders	5.0 [4–6]	8.0 [8–9]	+3.00 [2.00–5.00]	0.0010
Managing anorexia/restrictive EDs	3.0 [2–4]	6.0 [6–7]	+3.00 [2.00–5.00]	0.0164
Managing binge eating disorders	2.0 [2–4]	7.0 [4–7]	+3.00 [2.00–6.00]	0.0090

Comfort rated on a 1–10 scale. Wilcoxon signed-rank test, *n* = 11. All domains significant at *p* < 0.05. 95% CIs are bootstrap-based. *r* = rank-biserial correlation effect size.

### Secondary outcomes: screening practices and identification rates

Secondary outcomes included self-reported estimates of the percentage of patients screened for eating disorders and the percentage of patients who screened positive (Table 2). Prior to the intervention, no participants reported using any standardized eating disorder screening tool. Baseline self-reported screening frequency was low, with physicians screening a median of 5% (IQR 5%–15%) of their patients for eating disorders in the prior three months. Baseline triggers for screening patients for disordered eating included concerning responses during HEADSS (Home, Education/Employment, Activities, Drugs, Sexuality, Suicide/depression) assessments (96%), changes in weight/growth (92%), parental concerns (84%), abnormal physical examination findings (72%), or activity/employment concerns (40%).

**Table 2:** Screening and diagnosis rate estimates: pre- vs. post-intervention (Wilcoxon signed-rank, *n* = 11).

Outcome	Pre Mean	Post Mean	Mean Change	*p*-value	Significant
% Patients Screened (*n* = 11)	5% [IQR 5%–15%]	45% [IQR 15%–75%]	+40 pp	0.0039	Yes
% Screened Positive/Diagnosed (*n* = 11)	5% [IQR 5%–5%]	15% [IQR 5%–15%]	+0 pp	0.0625	No

pp = percentage points. ID 030 excluded from all analyses (self-reported non-attendance). Estimates based on bucket midpoints of self-reported response bins. 95% CIs are bootstrap-based. *r* = rank-biserial correlation effect size.

Following the intervention, self-reported screening frequency increased markedly with the median estimated percentage of patients screened rising from 5% (IQR 5%–15%) to 45% (IQR 15%–75%), an estimated median change of +40 percentage points (95% CI 10–60; *p* = 0.0039, *r* = 1.000).

In contrast, the estimated percentage of patients who screened positive for disordered eating did not change significantly following intervention. The median remained at 5% in both the baseline (IQR 5%–5%) and follow-up (IQR 5%–15%) surveys, corresponding to an estimated median change of 0 percentage points (95% CI 0–10; *p* = 0.0625; *r* = 1.000). This non-significant result likely reflects a floor effect, as nearly all participants reported screening fewer than 10% of patients positive at baseline, leaving limited room to detect significant change.

### Screening tool use and perceived impact

At six months, 65% of respondents reported using the.EDscreen tool at least once (vs 0% at baseline), and 87% rated the interventional curriculum as beneficial. Qualitative feedback, collected via free-response items on the six-month follow-up survey (Survey 3), indicated improved confidence initiating conversations with patients and families, a more structured approach to screening and escalation of care, and greater perceived support in the outpatient setting management of eating disorders.

## Discussion

This study demonstrates that an educational curriculum paired with an EMR-integrated screening tool was associated with significant improvements in physician self-reported comfort with eating disorder screening and management over a six-month period. Across all three domains—screening for eating disorders, managing restrictive eating disorders, and managing binge eating disorders—physicians showed statistically significant gains in comfort (all *p* < 0.05, Wilcoxon signed-rank, large effect sizes), supporting our primary hypothesis. These findings are consistent with prior work demonstrating that ED-focused didactic and clinical trainings are associated with greater comfort and confidence in diagnosing and treating eating disorders among physician trainees (5, 6). Despite growing recognition of the need for physician education in this area, training opportunities remain limited: a national survey of 637 residency programs found that 81% did not offer required or elective rotations focused on eating disorders, and fewer than a quarter of residents felt their training had adequately prepared them to address these conditions (6). The present study offers evidence that a targeted curriculum can meaningfully address this gap within the structure of an existing outpatient training program.

The magnitude of improvement was notable across all three provider comfort domains with an estimated median change of +3.00 points on a 10-point scale for each domain. Notably, baseline comfort scores in this cohort were consistent with those reported in prior studies of primary care providers: Lebow et al. found a mean screening confidence score of 5.53 (SD 2.09) among 60 pediatric and family medicine providers—comparable to our pre-intervention median of 5.0—and even lower confidence scores for management tasks (7). The greatest absolute gain was observed in the binge eating domain, which also had the lowest baseline comfort score, suggesting this was the area of greatest unmet educational need. This finding is consistent with existing evidence that preclinical medical education disproportionately emphasizes anorexia nervosa and bulimia nervosa, often leaving binge eating disorder, ARFID, and OSFED inadequately covered (6). The post-intervention IQR for screening comfort (8–9) was narrow compared to baseline (4–6), suggesting that the curriculum promoted convergence toward high, consistent comfort levels across the cohort, likely reflecting the standardizing effect of the screening tool as a shared clinical framework.

Qualitative feedback from the six-month survey reinforced these quantitative findings. The majority of respondents (87%) reported that the curriculum was beneficial, with common themes including increased confidence in approaching screening conversations, clearer guidance on escalation of care, and recognition of the relevance of eating disorder management to general outpatient pediatrics. Several physicians specifically highlighted the value of structured next steps for when disordered eating is identified—with close follow-up, and family-based conversations emerging as the most commonly reported responses. This aligns with findings from Lebow et al., in which primary care providers expressed strong interest in training and practical tools for eating disorder management but emphasized that interventions would be most helpful if they fit within the workflow and time constraints of the primary care setting (7). The screening tool was turned into an EMR-embedded dot phrase with this principle in mind.

Use of the screening tool was encouraging, with 65% of post-survey respondents reporting use of the tool at least once, compared to 0% at baseline. Screening rates increased significantly following the intervention (median +40 pp; *p* = 0.004), consistent with improved tool use translating into clinical behavior. However, qualitative responses revealed that a meaningful subset of physicians forgot the screening tool dot phrase was available, often relying instead on informal questions. Several respondents suggested that better workflow integration, such as embedding the screener as a disappearing prompt within the HEADSS assessment or displaying reminders at clinic workstations, would improve uptake. This mismatch between knowledge and behavior is well-documented in the eating disorders literature. Brown et al. found that even among residents who had received eating disorder training in their residency program, a meaningful number still reported rarely or never screening for eating disorders in practice, highlighting that education alone does not guarantee consistent clinical behavior (5). These findings suggest that improving eating disorder screening requires both building provider confidence and embedding tools within existing clinical workflows.

Several alternative explanations for the observed improvements in physician comfort should be considered. Because the study lacked a concurrent control group, it is possible that simply being observed and surveyed about eating disorder screening (a Hawthorne-type effect) heightened participants' attention to the topic independent of the curriculum or screening tool itself. Similarly, secular trends, such as rising national awareness of adolescent mental health and eating disorders following the COVID-19 pandemic, or concurrent continuing medical education unrelated to this study, could have contributed to the observed gains. The consistency of the effect across all three comfort domains and its alignment with the specific content of the curriculum (e.g., the largest gains in the binge eating domain, an area the curriculum specifically addressed) argue against secular trends as the sole explanation, but a controlled or stepped-wedge design would be needed to more rigorously isolate the intervention's independent effect.

Broader implementation of this model would need to account for differences across clinical settings and provider types. CHLA AltaMed is a single, resource-supported academic-affiliated outpatient clinic; community-based practices with fewer administrative resources or less access to EMR customization support may face greater barriers to building and maintaining an equivalent EMR-integrated screening tool. Likewise, this cohort was limited to physicians (residents, fellows, and attendings); extending this curriculum to other provider types who conduct well-child visits, such as nurse practitioners and physician assistants, would broaden its reach but has not been evaluated here and may require adaptation of curriculum content or delivery format to fit different training backgrounds and workflows.

This study has several limitations that should be considered when interpreting these findings. First, the sample size was small (*n* = 11 matched pairs after exclusion of one non-attendee), which limits statistical power and the generalizability of the results. Relatedly, both study enrollment and completion of both surveys required for the paired analysis were voluntary, raising the possibility of selection bias: physicians who chose to participate and to complete follow-up may have been more motivated or already more engaged with eating disorder care than non-participants or non-completers, which could inflate the observed improvements relative to what would be seen with universal participation. Second, the study used a pre-test/post-test design without a concurrent control group, making it difficult to attribute observed changes solely to the intervention rather than to other factors such as clinical experience accumulated over the study period. Third, all outcome measures were self-reported, introducing the potential for social desirability bias and recall inaccuracy, a limitation shared by much of the existing literature on physician comfort with eating disorders. Relatedly, this study did not capture objective, patient-level outcomes such as referral rates to specialty eating disorder care, time to treatment initiation, or clinical trajectory; without these data, it is not possible to determine whether improved physician comfort and self-reported screening translated into meaningful downstream benefits for patients, and future work should incorporate such measures (5, 7). Fourth, curriculum participation was voluntary and untracked, meaning that exposure to individual components varied across participants and could not be correlated with specific outcomes. Fifth, the study was conducted at a single site (CHLA AltaMed), which limits generalizability to other outpatient pediatric settings with different patient populations or training structures. Finally, although a statistically significant increase in self-reported screening rates was observed (*p* = 0.004), the percentage of patients screening positive did not reach significance (*p* = 0.063), likely due to a floor effect at baseline. Lastly, all behavioral outcome estimates, including use of the.EDscreen dot phrase, were provided by participants rather than extracted from the EMR via structured query, limiting the precision and objectivity of these secondary outcomes. In particular, dot phrase usage was assessed by self-report rather than by structured query of the EMR itself, which may not accurately reflect true utilization rates. Finally, this study did not include a balancing measure to assess the potential burden of additional screening responsibilities on physician workload; the introduction of additional mental health-related screening has been associated with increased burnout among pediatricians, and future iterations of this work should incorporate such a measure (8).

Despite these limitations, this study offers several important strengths. The intervention was designed and implemented within an existing clinical training program, demonstrating real-world feasibility without requiring dedicated research personnel or external funding for curriculum delivery. The use of an EMR-integrated dot phrase represents a practical, scalable model that could be adapted at other outpatient sites. Additionally, this study population is one in which improving eating disorder detection has important clinical and equity implications. The CHLA AltaMed patient population is predominantly publicly insured and Latinx, groups that experience documented disparities in eating disorder identification and access to care. By training general pediatricians who care for this community, this intervention directly addresses a critical gap in the continuum of eating disorder care for underserved populations.

Future directions should address the key barriers identified in this study. Integrating the screening tool directly into the HEADSS assessment workflow, as suggested by multiple respondents, would reduce reliance on provider recall and improve screening consistency. Formal incorporation of the curriculum into the adolescent medicine elective rotation would ensure utilization of the curriculum lectures for all residents rather than depending on voluntary attendance. EMR-based chart review in future studies would allow for objective, independent verification of the self-reported changes in screening and its impact on patient-centered outcomes such as earlier detection and improved functional and psychosocial trajectories. Finally, expansion to additional AltaMed and CHLA affiliate sites, along with development of multilingual patient-facing educational materials, would extend the reach of this intervention to broader populations.

In conclusion, these findings support the value of structured, curriculum-based education in improving physician comfort in identifying and managing eating disorders in the outpatient pediatric setting and highlight the potential of EMR-integrated tools to standardize screening.

## Data Availability

The original contributions presented in the study are included in the article/Supplementary Material, further inquiries can be directed to the corresponding author/s.
